# Mechanisms of triple‐negative breast cancer extravasation: Impact of the physical environment and endothelial glycocalyx

**DOI:** 10.1096/fj.202400380R

**Published:** 2024-07-01

**Authors:** Chinedu C. Okorafor, Sanjana Shastri, Ke Wen, Eno E. Ebong

**Affiliations:** ^1^ Department of Chemical Engineering Northeastern University Boston Massachusetts USA; ^2^ Department of Behavioral Neuroscience Northeastern University Boston Massachusetts USA; ^3^ Department of Bioengineering Northeastern University Boston Massachusetts USA; ^4^ Department of Neuroscience Albert Einstein College of Medicine New York New York USA

**Keywords:** cancer cell extravasation, endothelial cells, extracellular matrix, glycocalyx, tissue stiffness, vascular flow

## Abstract

Cancer metastasis is the leading cause of death for those afflicted with cancer. In cancer metastasis, the cancer cells break off from the primary tumor, penetrate nearby blood vessels, and attach and extravasate out of the vessels to form secondary tumors at distant organs. This makes extravasation a critical step of the metastatic cascade. Herein, with a focus on triple‐negative breast cancer, the role that the prospective secondary tumor microenvironment's mechanical properties play in circulating tumor cells' extravasation is reviewed. Specifically, the effects of the physically regulated vascular endothelial glycocalyx barrier element, vascular flow factors, and subendothelial extracellular matrix mechanical properties on cancer cell extravasation are examined. The ultimate goal of this review is to clarify the physical mechanisms that drive triple‐negative breast cancer extravasation, as these mechanisms may be potential new targets for anti‐metastasis therapy.

## Graphical abstract

TNBC extravasation into the secondary tumor environment, emphasizing (1) vascular flow, (2) subendothelial matrix stiffness, and (3) physical endothelial glycocalyx barrier. Focusing on these mechanical aspects driving extravasation, a critical metastasis step, the review contributes fresh perspectives to understanding TNBC metastasis, potentially revealing new therapeutic targets.

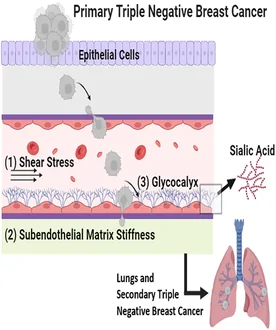

**Abbreviations**
BM: Basement Membrane
CS: Chondroitin Sulfate
CTCs: Circulating Tumor Cells
ECs: Endothelial Cells
EM: Extracellular Matrix
GCX: Glycocalyx
HA: Hyaluronic Acid
HS: Heparan Sulfate
SA: Sialic Acid
TNBC: Triple Negative Breast Cancer
WGA: Wheat germ agglutinin

## 1 INTRODUCTION: UNCOVERING THE MECHANISMS DRIVING CANCER EXTRAVASATION

Cancer is one of the deadliest diseases, with breast cancer being the fifth leading cause of cancer death.^1^ Within the breast cancer category, triple‐negative breast cancer (TNBC) accounts for about 30% of all cancer deaths.^2^ TNBC is an aggressive breast cancer with no estrogen and progesterone receptors. It possesses very little human epidermal growth factor receptor 2 (HER2) protein.^2^ Annually, approximately 170 000 cases are diagnosed worldwide.^3^ It is a cancer known to be highly metastatic and is commonly diagnosed for women at an earlier age (<40 years).^4^, ^5^ Usually, mammogram procedures are recommended for 40 years old and later, leading to late diagnosis. This late diagnosis may be responsible for its spread beyond the primary site. Once the primary cancer has spread to other organs, it is diagnosed as stage IV cancer (metastatic cancer) with a very low five‐year survival rate of 11%.^6^ Cancer metastasis has been proven as the primary cause of cancer‐related deaths, as indicated by several studies including one population‐based study that attributed at least two‐thirds of cancer‐related deaths to metastasis.^7^, ^8^, ^9^

Cancer metastasis is a phenomenon in which cancer cells expand beyond their primary residence by traveling to new body areas through lymph nodes or blood vessels (Figure 1).^10^ Focusing on blood vessels, the primary tumor becomes vascularized, leading cancer cells to detach and penetrate through layers of blood vessels into the bloodstream (Figure 1).^11^ Some blood circulating tumor cells (CTCs) die while others survive and go through a process of extravasation, which begins with adhering to adhesion molecules of endothelial cells (ECs) that may be nearby or distant from the primary site (Figure 1).^12^ The survivors then transmigrate across the vessel walls composed of endothelium and basement membrane (BM) and attach themselves to interstitial matrix at secondary sites. Cancer cells that survive extravasation at the secondary sites will then either die, stay in dormancy, or proliferate and form tumors through colonization.^13^ When TNBC metastasizes, it often spreads to lungs, liver, bone, and brain, with lung being the most common first site of incidence at 41%.^11^, ^14^

**FIGURE 1:**
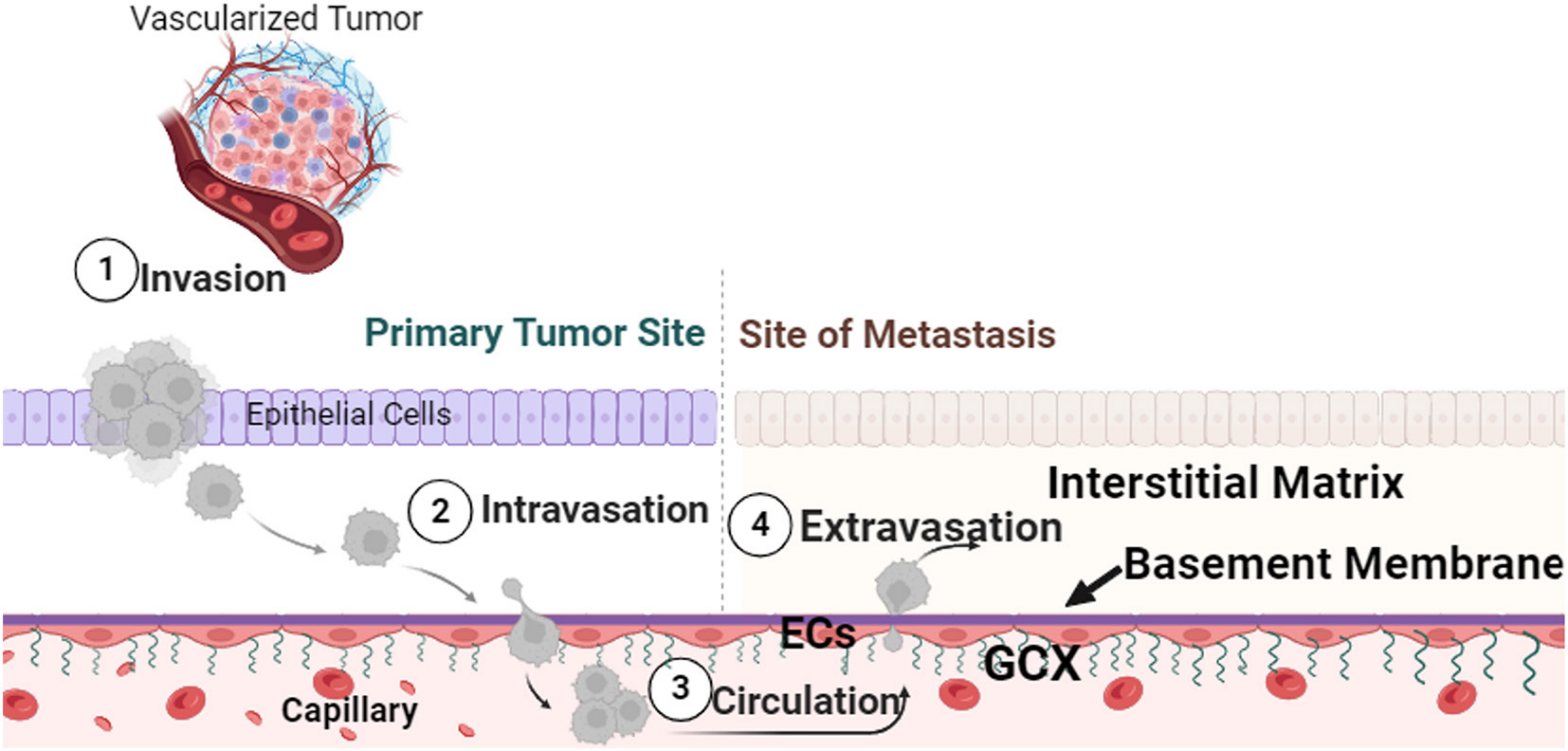
Overview of metastatic cascade. (1) Vascularization of tumor causes tumor cells to detach and migrate toward main vessels, subsequently (2) invading nearby blood vessels to (3) enter the blood circulation. Circulating tumor cells eventually undergo (4) extravasation through capillary walls and land in a secondary site for potential colonization. Adapted from “Overview of Metastatic Cascade,” by BioRender.com (2024). Retrieved from https://app.biorender.com/biorender‐templates.

Lungs are common sites of metastasis probably due to their fibrotic nature. This fibrosis may be from either lifestyle (smoking), radiation treatment of primary tumor, or “pre‐metastatic niche” priming from primary tumor.^15^, ^16^, ^17^ Lung fibrosis is a disease that occurs when lung tissue becomes scarred from an excess of extracellular matrix (ECM) deposition in the interstitial matrix resulting in heightened stiffness (20‐100 kPa) compared to normal lung matrix (1–5 kPa).^18^ Studies have shown that a fibrotic lung promotes the metastatic seeding of TNBC.^17^, ^19^, ^20^, ^21^, ^22^, ^23^ These studies have focused on molecular and chemical mechanisms by identifying the chemokines (fibronectin‐1, secreted phosphoprotein‐1), growth factors (TGF‐β), and ECM protein composition (LOX‐induced collagen I crosslinking) involved in promoting this seeding in lungs^20^, ^21^, ^24^ according to Dr. Pagat's “seed and soil” concept. This concept hypothesized that the secondary tumor microenvironment, “the soil,” must be compatible with the CTC, “the seed,” in order for metastasis to take place.^25^, ^26^ However, these studies focus on the final colonization step of the metastatic cascade and not the delivery of CTCs to secondary tissue sites, specifically the cancer extravasation step. Without cancer cells' adherence and extravasation out of vessel wall, cancer cells will die in circulation. It is important to understand more of the mechanisms driving cancer cells to be delivered to this organ site.

A popular theory often proposed to explain CTCs' delivery to these secondary sites is the “mechanical trapping theory.” This can happen in capillaries, which are already critical for CTC extravasation due to slow blood flow velocities and thin walls, that facilitate transvascular transport of CTCs and other biological substances from blood into tissue (Figure 1).^27^ Capillaries also have small diameters that often range from 5 μm to 10 μm,^28^, ^29^, ^30^ which are smaller than CTCs' size (TNBC's diameter 12.4 ± 2.1 μm)^31^ and are characterized by their many branches. In the trapping theory, capillaries' small diameters and many branches allow CTCs to be physically trapped in the blood vessel for extravasation. However, physical trapping of CTCs is not always sufficient for CTC attachment and extravasation.^32^, ^33^ Otherwise, the lung would be the only site of metastasis since it is the first organ downstream of the mammary gland and CTCs would not be able to escape and travel to different organs.^34^, ^35^ In addition to trapping, or even in the absence of trapping, active adhesion between CTCs and the endothelium under flow is required for initiation of the extravasation process.^32^, ^36^, ^37^

The adhesion barrier consists of a number of possible components that may be involved in regulating CTCs' interaction with its potential secondary site. Within capillaries, these components include endothelial glycocalyx (GCX), ECs, and BM, that may vary depending on the capillary type. There are three types of capillaries—continuous, fenestrated, and sinusoidal. As it has been noted that TNBC metastasizes to lungs, liver, bone, and brain, it is important to mention that liver and bone marrow capillaries are sinusoidal, while lung and brain capillaries are continuous. For sinusoidal capillaries, ECs are filled with large fenestrae (pores) and few tight junctions in between the cells, leaving large openings (as large as 180 nm for liver) between the ECs.^38^ For liver capillaries, there is no basement membrane underneath the fenestrated endothelium, which increases the overall permeability of the vessel, although there are pericytes lying between ECs and hepatocytes.^39^ Of the capillaries, continuous capillaries are the least permeable with ECs forming a continuous lining and adjacent ECs joining together via cell–cell adhesions. These cell–cell adhesions mediate vascular permeability and form a barrier against CTC extravasation. Tight junctions (i.e., claudins and occludins), in particular, form an impermeable barrier toward macromolecules.^38^ In addition, both lung and brain microenvironment have a continuous BM underneath ECs surrounded by supportive cells such as pericytes and, for the brain, astrocytes. Due to their tight junctions and continuous BM, lungs and brain form a strong barrier to cancer extravasation over the other organs, however these organs are popular sites of metastasis for TNBC. The “carbohydrate‐rich” coating on ECs, GCX, may play a critical role, along with the surrounding mechanical environment, in mediating the adhesion that is necessary for initiating extravasation. However, the capillary EC GCX is understudied.

Once adhesion occurs, CTC delivery to secondary sites is not complete without extravasation. In studies that have considered the mechanical environment, the CTC extravasation process and its resulting migration through interstitial matrix of the secondary tissue site has been shown to depend on the mechanical forces due to shear stress from both bulk blood flow and interstitial fluid.^11^, ^36^, ^37^, ^40^, ^41^, ^42^ Bulk blood flow regulates both the adhesion between ligands of CTCs and receptors found on the endothelium of the vessel wall and CTCs' extravasation into surrounding tissue.^42^, ^43^, ^44^ Once CTC extravasation occurs, migration of CTCs through the interstitial matrix is influenced by interstitial flow^42^, ^45^ and stiffness of the BM and interstitial matrix, subendothelial extracellular matrix (ECM) (Figure 2).^46^, ^47^, ^48^ Notably, the bulk blood flow and matrix stiffness are all expected to influence the GCX barrier structure and permeability function.^49^, ^50^, ^51^, ^52^, ^53^ Understanding the structure and potential role of the mechanically regulated GCX during CTC extravasation will help identify new potential targets for anti‐metastasis therapy.

**FIGURE 2:**
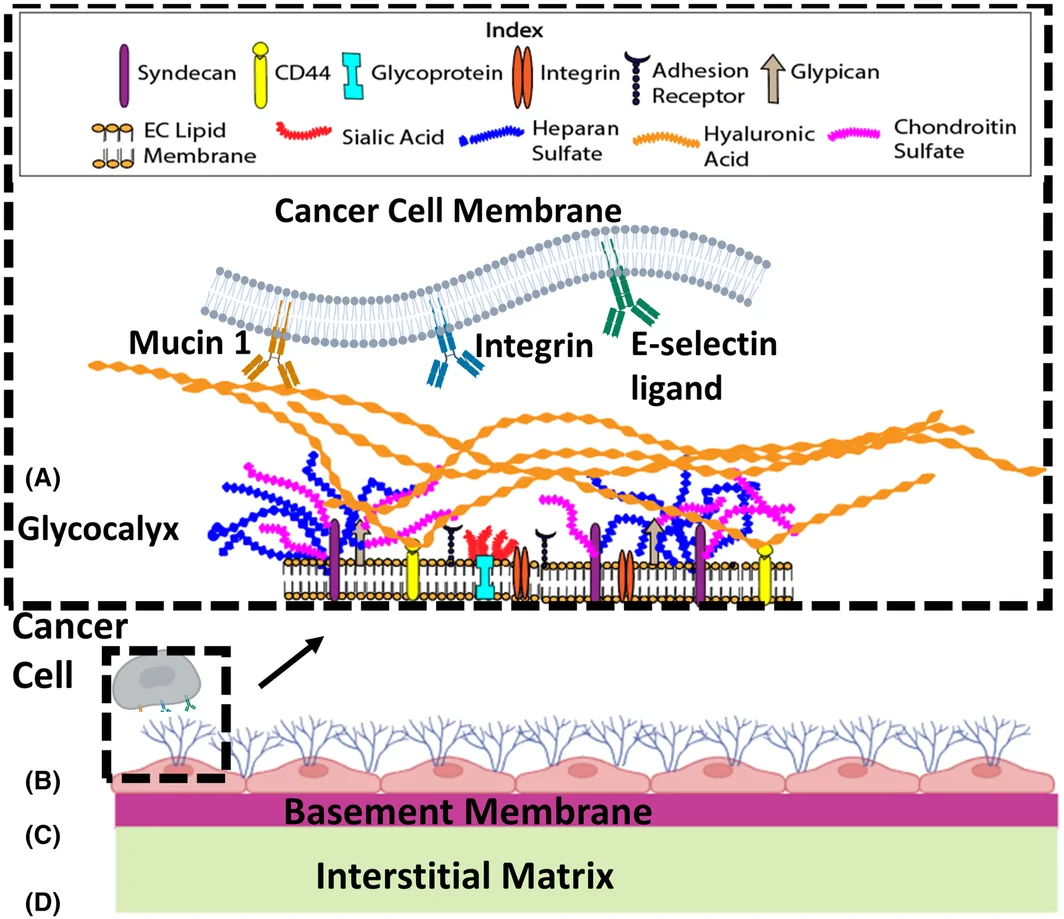
Capillary endothelial glycocalyx may be the critical under‐explored vascular barrier that regulates cancer cells interactions with endothelium. (A) On the luminal side of endothelial cells lies a negatively charged layer that covers the endothelium surface called the glycocalyx (GCX). This structure is composed of glycosaminoglycans (GAGs) such as heparan sulfate (HS), chondroitin sulfate (CS), hyaluronic acid (HA), and sialic acid (SA) connected to cell membrane bound core proteins and receptors such as syndecan, glypican, CD44, and glycoproteins. The GCX is the first vascular barrier component that circulating cancer cells face along the inner capillary wall, so it may play a key role in mediating cancer metastasis. The GAG chains and soluble plasma components cover the adhesion molecules (integrin, selectins, and immunoglobulins) of the endothelium by preventing them from interacting with cancer cells' ligands (mucin 1, integrin, and E‐selectin). (B) The endothelial cell (EC) monolayer is the second layer of the vascular barrier. (C) Underneath the ECs lies the third layer, a supportive basement membrane, that cancer cells must cross through to escape the vessel to land on (D) the interstitial matrix in the secondary site. Created with BioRender.com.

This paper reviews some of the studies on the role that mechanical factors such as vascular shear stress and matrix stiffness play in guiding cancer extravasation, primarily considering the involvement of the endothelial GCX structure. The paper will conclude with a discussion of potential future studies.

## 2 ROLE OF MECHANICAL ENVIRONMENT IN CANCER EXTRAVASATION

The mechanical environment's effect on cancer extravasation is greatly understudied, especially with respect to the flow dynamics (vascular shear stress) and contact forces (i.e., stiffness) experienced by ECs lining the inner capillary wall that affects its barrier function against cancer extravasation (Figure 3). Once CTCs approach the endothelium wall, there is a struggle for dominance between the bulk flow vascular shear stress and the adhesive strength of CTCs to the endothelium wall.^49^ As shear stress varies from organ to organ, the possibility that this variable plays a key role in CTCs' metastasizing potential during circulation has been explored.^37^ Follain et al. found that under specific blood flow velocities, ECs undergo remodeling.^36^ In the endothelial remodeling process, ECs form tight connections with each other and engulf the arrested CTCs (“pocketing step”), which quickly induces extravasation. For laminar blood flow velocities that ranged from 400 to 600‐μm/s, CTCs were rapidly surrounded by ECs and extravasated, while reduced blood flow (<400 μm/s) slowed down the endothelial remodeling (“pocketing step”), which impaired/hindered successful extravasation.^24^ Endothelial remodeling was driven by vascular endothelial growth factor 2 (VEGFR2) signaling, downstream of vascular flow.^54^ Once VEGFR2 is activated, there are two possible signaling pathways it could take to induce endothelial remodeling via actin reorganization: Cdc42/p38/MAPKAPK/HSP27 or SRC/PI3K/PIP3/RAC (Figure 3).^55^, ^56^ In addition, scientists have also started to determine what shear stresses favor CTC adhesion. In the Follain et al. study, highly metastatic breast cancer cells were injected into mouse hearts and the research team examined the CTCs' arrest, extravasation, and growth in the brain. They found that in capillaries where extravasation predominantly occurred due to endothelial remodeling, the average flow velocity of blood was 628 ± 46 μm/s, and the shear stress ranged from 5 to 7 dyn/cm^2^.^36^, ^37^ There is a need for further studies to determine whether these shear stress values result in efficient extravasation in other organs and whether the endothelial remodeling process also occurs in human organs.

**FIGURE 3:**
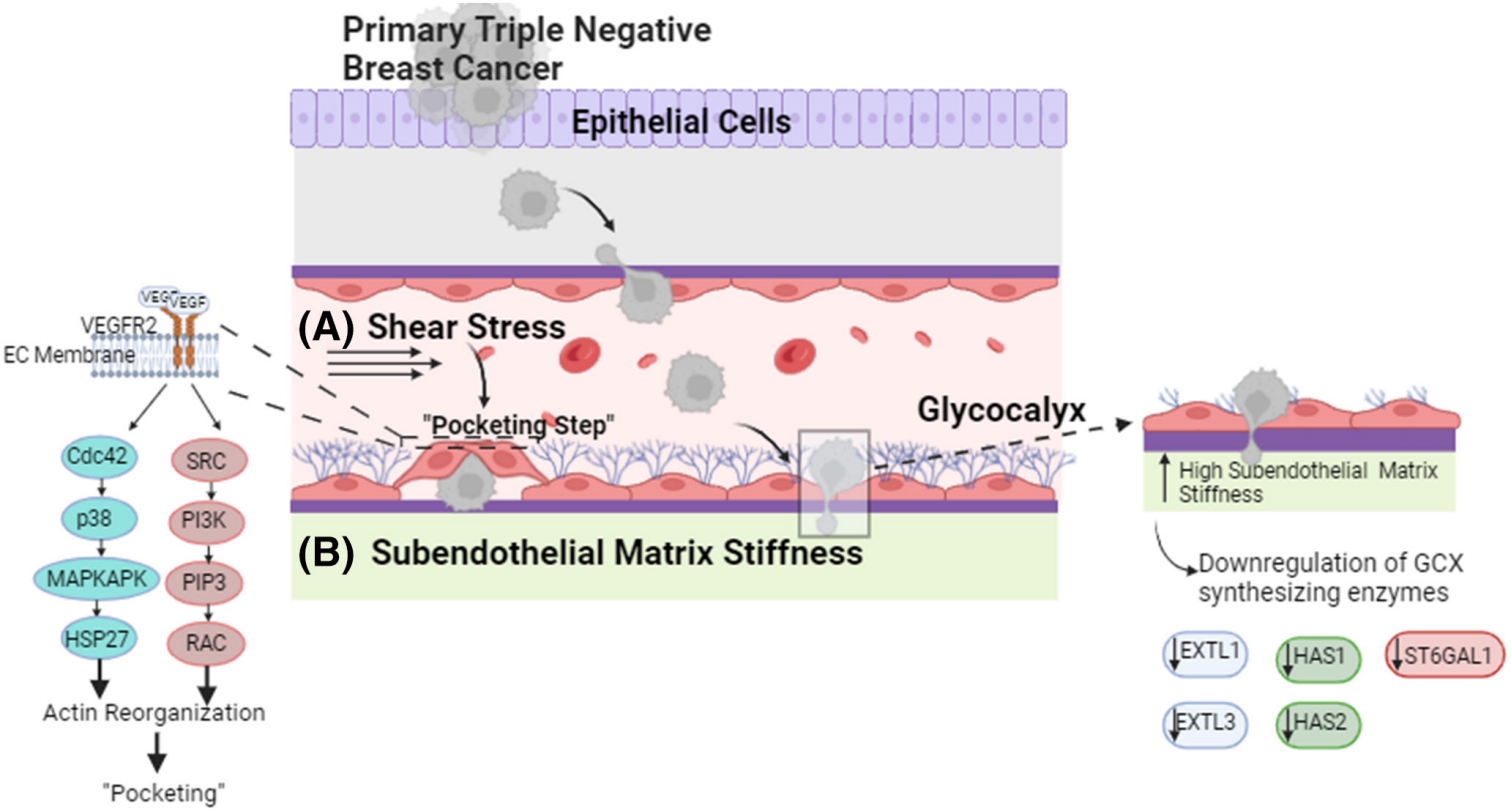
Vascular hypothesis of mechanical environment's impact on cancer cell extravasation. (A) Vascular shear stress induces upregulation of vascular endothelial growth factor receptor 2 (VEGFR2) on ECs, which remodel to create a pocket for metastasizing cancer cells. Once VEGFR2 is activated, there are two possible signaling pathways it could take to induce remodeling via actin reorganization: Cdc42/p38/MAPKAPK/HSP27 or SRC/PI3K/PIP3/RAC.^55^, ^56^ (B) High subendothelial matrix stiffness is hypothesized to reduce GCX expression through a downregulation of GCX synthesizing enzymes such as EXTL1/EXTL3 [HS synthesis], HAS1/HAS2 [HA synthesis], and ST6GAL1 [α2,6‐linked SA synthesis].^53^ The reduced GCX expression allows cancer cells to attach to endothelium and transmigrate through the subendothelial matrix. Created with BioRender.com.

The impact of subendothelial ECM stiffness's role on controlling cancer extravasation is mostly unknown with only two studies probing its role. Matrix stiffness may be a key mediator of cancer extravasation because, first, the matrix properties affect cancer cell function, second, the matrix supports endothelium and affects its barrier function, and third, the matrix is the immediate next medium that cancer cells must enter after passing through the endothelium. Azadi et al. investigated the effect of matrix stiffness by preconditioning human breast cancer cells, specifically MDA‐MB‐231 cells, on several matrix stiffness conditions (1.1 ± 0.2 MPa, 14.3 ± 3.2 kPa, and 5.2 ± 1.3 kPa) for 24 h prior to injecting them into a human umbilical vein EC (HUVEC) cultured transwell system under static conditions.^57^ Cancer cells preconditioned on stiff gels showed a greater extravasation (40 ± 12% for stiff group and 30 ± 6.87% for soft group) and migration distance (42% increase compared to soft group).^57^ The increase in extravasation and migration distance correlated to an increase in MMP‐9 secretion which directly degrades ECM. Although this study showed how the initial environment (primary tumor)'s mechanical environment affected cancer extravasation, the subendothelial matrix stiffness role was not studied. Hamilla et al. examined the effect of subendothelial matrix stiffness on cancer cells' incorporation, a process in which cancer cells physically displace ECs' monolayer and spread on BM.^58^ In their study, TNBCs' incorporation stayed the same for HUVECs cultured on matrices of varying stiffness conditions (0.87, 280 kPa, and glass (50 MPa)).^58^ It was observed that substrate stiffness did not affect the incorporation stage of cancer extravasation, though it is possible that it could affect the amount of cancer cell attached to endothelium and its resulting migration through matrix through GCX regulation. Hamilla's study also did not incorporate flow which may affect endothelial barrier function and CTCs' potential attachment and incorporation.

Interstitial flow is another mechanical factor driving cancer migration. Interstitial flow is characterized as the fluid flow across the blood vessel wall and into the interstitial matrix. Compared to bulk blood flow, interstitial flow moves at a much slower velocity (0.1–4 μm/s) because of resistance from the ECM. Previous studies have found that interstitial flow enhanced chemotaxis‐induced migration of cancer cells, with cancer cells moving in the direction of flow through the matrix, and that interstitial flow increased overall cancer cell migration and transmigration speed through the matrix compared to static condition.^59^, ^60^ In terms of CTC extravasation, only one study done by Hajal et al. has addressed the function of interstitial flow in CTC extravasation by subjecting CTCs to both physiological luminal flow and trans‐endothelial flow during CTC attachment to the endothelium.^42^ In an in vitro microvascular network model, they injected MDA‐MB‐231 cells under luminal flow (1.5 dyn/cm^2^) coupled with transendothelial flow (1.24 and 2.14 μm/s).^42^ They determined that CTC extravasation efficiency is mainly a result of luminal flow (3‐fold increase compared to static) and not due to interstitial flow.^42^ However, interstitial flow increased the speed of extravasation and allowed CTCs to remain near the vasculature (“vessel co‐option”) for potential metastatic colonization after extravasation due to extravasated cells moving at several different angular directions and no singular direction down the matrix.^42^ To explain CTCs' enhanced extravasation under flow, they hypothesized that the endothelial tight junctions reorganized, and transcription factors were activated on cancer cells enhancing cancer cell extravasation. However, they did not find any significant changes in endothelial cell morphology during extravasation under flow. Another possibility for this behavior could be the mechanical environment's effect on surrounding microenvironment, specifically the endothelial GCX.

## 3 INFLUENCE OF MECHANICAL FORCES ON ENDOTHELIAL GCX BARRIER FUNCTION

### 3.1 GCX composition, structure, and barrier function relevant to TNBC extravasation

The endothelial GCX is a negatively charged, carbohydrate‐rich layer that covers the entire endothelium surface and is most abundant on the luminal surfaces of ECs. It is made up of heparan sulfate (HS), hyaluronic acid (HA), and chondroitin sulfate (CS) glycosaminoglycans (GAGs), as well as negatively charged sialic acid (SA) (Figure 2).^61^ These components are interlinked with proteoglycan (syndecans, glypicans) and glycoprotein backbone molecules that connect the GCX to the endothelium (Figure 2).^61^ Since the structure is found to be abundant on the luminal surfaces of ECs, it acts as a protective barrier against circulating blood components. The negatively charged GCX layer repels and limits the permeability of numerous large blood components (including CTCs) while allowing albumin and other small molecules to pass through.^61^ GAG chains that extend into the vascular lumen and their associated soluble plasma proteins shield EC adhesion molecules that lie at the basal portion of the GCX. EC adhesion molecules, such as selectins, integrins, and immunoglobulin super family (intercellular adhesion molecule [ICAM‐1, ICAM‐2], vascular cell adhesion molecule [VCAM‐1], and platelet/EC adhesion molecule 1 [PECAM‐1]), are involved in EC recruitment and binding to CTCs from the blood stream to the endothelium.^61^, ^62^, ^63^ In the extravasation process, CTCs roll along the endothelium by interacting with selectins and adhere firmly and extravasate out of the vascular wall through integrin and immunoglobulin super family interactions. As these adhesion molecules are hidden under the GCX, CTCs' adhesion to the endothelium can reduce significantly when the GCX is intact.^64^, ^65^

Previous studies have shown that HS and HA expressed on large vessel ECs such as human umbilical vein endothelial cells (HUVECs) limited cancer cell attachment and extravasation.^44^, ^66^ HS and HA are known to be major components of the GCX for these cells.^61^ Due to these components being major constituents, Offeddu et al. observed that when HS, HA, and CS were individually removed by enzymatic degradation (heparinase, hyaluronidase, and chondroitinase), TNBC extravasation efficiency increased significantly with HS and HA degradation while CS degradation showed no change.^44^ A few studies have shown that strengthening the GCX inhibited TNBC extravasation. Cai et al. attempted to preserve GCX barrier integrity by pretreating ECs with orosomucoid, an α1‐acid glycoprotein known to boost the negative charge and organization of GCX structure.^66^, ^67^, ^68^ They found that orosomucoid increased HS expression which reduced tumor cell adhesion by 46% compared to the control.^66^ Zhang et al. showed that sphingosine‐1‐phosphate (S1P), a sphingolipid carried by albumin, also protected GCX structure.^69^ They determined that S1P protected GCX by inhibiting matrix metalloproteinases (MMP) shedding of HS, which resulted in the attenuation of cancer cell adhesion (39% of the control).^69^ Therefore, the presence of HS and HA on ECs protects the hidden EC adhesion molecules from interacting with CTCs and provides a strong barrier against cancer extravasation.

However, negatively charged SA residue's role in mediating this process is unclear with earlier studies showing contradiction. Mensah et al. performed two in vitro studies that examined interactions between SA‐degraded ECs (rat fat pad ECs or HUVECs) with TNBC cells (specifically 4T1 cells, which is a mouse model) for 30 min under static conditions and 1 h under flow conditions.^64^, ^65^ The decreased ECs' SA expression resulted in a significant increase of cancer cell attachment and initial migration through the endothelium.^64^, ^65^ In Zemljič‐Jokhadar et al.'s in vitro study the SA‐degraded HUVECs interacted with TNBC cells (specifically MDA‐MB‐231 cells, which is a human model) for 20 min under static and 15 min under flow. The decreased ECs' SA expression did not change TNBC attachment from the untreated control.^70^ The differences in TNBC behavior could be due to the length of exposure time of CTCs to SA‐degraded ECs as cancer extravasation occurs as early as 30 min after CTC introduction to the vasculature.^71^ The difference could alternatively be due to the cancer cell type (mouse 4T1 vs human MDA‐MB‐231) having different binding ligands for endothelium. More work needs to be done in clarifying SA's role as a mediator of human TNBC (MDA‐MB‐231) extravasation to get a better understanding of how GCX composition controls this process.

### 3.2 Regulation of endothelial GCX composition by mechanical environment

Along the inner capillary wall, the endothelial GCX experiences several mechanical factors from its underlying matrix and blood flow. Studies have looked at endothelial GCX regulation by mechanical environment under the context of atherosclerosis with a focus on the effect of vascular shear stress induced by blood flow or subendothelial matrix stiffness.^51^, ^72^, ^73^, ^74^, ^75^, ^76^ High vascular shear stress has been shown to increase GCX expression for macrovascular ECs. In Gouverneur et al.'s study, HUVECs exposed to a uniform vascular shear stress of ~10 dyn/cm^2^ for 24 h had a threefold increase of HA incorporated in the GCX when compared to static‐conditioned ECs.^77^ Zeng's study in which rat fat pad ECs were exposed to a uniform shear stress of 15 dyn/cm^2^ for 24 h showed a significant increase in HS, CS, glypican‐1, and syndecan‐1 expression.^50^ Koo et al. also showed that a 7‐day exposure to pulsatile shear stress (15–20 dyn/cm^2^) for HUVECs led to a 80.6 ± 33.0% increase in HS expression compared to static control and a 464.7 ± 15.6% increase in HA expression relative to the static control.^72^ The mechanism behind several GCX components synthesis due to high laminar shear stress remains largely unknown. A recent study done by Wang et al. investigated shear stress and HA synthesis by exposing HUVECs to a laminar shear stress of 10 dyn/cm^2^ for 4–7 days.^49^ They found that hyaluronan synthase 2 (HAS2), an enzyme that synthesizes HA, increased significantly under flow and that its production was highly dependent on Kruppel‐like factor 2 (KLF2) expression. When KLF2 was inhibited, it prevented the laminar shear induced increase of HA.^49^

Additionally, low shear stress (<5 dyn/cm^2^) has been shown to inhibit GCX expression. In Koo et al. exposed HUVECs to an “atheroprone” disturbed flow environment (oscillating, low vascular shear stress) which resulted in GCX shedding with a HS reduction of 28.5 ± 10.2% compared to static conditions.^72^ The HS reduction primarily came from syndecan‐1, a HS proteoglycan, downregulating its expression in an “atheroprone” flow environment. Low shear stress (2 dyn/cm^2^) was also found to promote HYAL2, an enzyme that degraded HA, which resulted in a significant reduction of HA expression for HUVECs.^78^ To prove that endothelial GCX's strong response to various shear stress environments also occurs in vivo, Van den Berg et al. examined the GCX dimensions of the “atheroprotective” common carotid artery and compared its dimensions to “atheroprone” sinus region of the inner carotid artery. They determined that GCX thickness has been greatly reduced in the disturbed flow region (73 ± 36 nm) when compared to the uniform flow region (398 ± 174 nm).^74^ Thus, the flow environment experienced by ECs heavily influences its GCX regulation.

In comparison to vascular shear stress, the effect of underlying matrix stiffness on GCX expression has been limited to three published studies to the best of our knowledge. ECs can sense up to several microns deep into a substrate, it likely senses the subendothelial matrix mechanical properties.^79^, ^80^, ^81^ In Mahmoud et al.'s studies, various types of ECs (HUVECs, rat fat pad ECs, bovine aortic ECs, mouse aortic ECs, and mouse brain immortalized microvascular ECs) were cultured on fibronectin‐coated polyacrylamide gels of varying stiffness (2.5–100 kPa) and fibronectin coated glass slides.^52^, ^53^ HS was suppressed for most cell types, except rat fat pad ECs, on glass slides compared to the more physiologically relevant matrix of 2.5 kPa.^52^, ^53^ Rat fat pad ECs and bovine aortic ECs showed HA inhibition on glass slides and glypican‐1 was suppressed on glass slides for all cell types except for bovine aortic ECs. Hamrangsekachaee et al.'s study in which HUVECs were cultured on gelatin‐methacrylate hydrogels of varying stiffness (2.5–10 kPa), found that whole GCX expression (wheat germ agglutinin lectin staining that labels SA and N‐acetylglucosamine, components of HS and HA) was not affected by increasing stiffness.^82^ However, the individual GCX components showed significant change with HS decreasing at higher stiffness and α2,6‐linked SA residue increasing at higher stiffness.^82^ It is still unknown the mechanisms driving individual GCX components (HS, HA, and SA) to be affected by the underlying matrix and why this regulation depends on cell type. More research needs to be done to see if human microvascular ECs' GCX components from metastatic sites (i.e., lung) will display similar behavior when regulated by subendothelial matrix stiffness.

Since the previous studies examining GCX regulation by stiffness and shear stress were in the context of atherosclerosis, there is a need to determine the role mechanically regulated GCX plays in guiding cancer extravasation. A recent study from our lab showed that disturbed flow induced GCX degradation led to a twofold increase of circulating metastatic breast cancer cells' attachment to HUVECs when compared to uniform flow conditions.^64^ Although the previous studies use of macrovessel ECs laid a foundation of knowledge on how endothelial GCX is impacted by its surrounding mechanical environment, GCX composition and structure varies within each organ microenvironment.^83^ EC GCX of various microvessels differ in structure and composition when compared to EC GCX of larger vessels and may respond differently to the same mechanical factors.^83^ There is a need to elucidate the mechanically regulated GCX composition of microvascular ECs from popular metastatic sites to help identify the relevant GCX components involved in promoting/preventing cancer extravasation.

### 3.3 Filling the gap: Vascular shear stress impact on lung and brain microvascular EC GCX expression

In literature, the characterization of the GCX of popular metastatic sites' capillaries (lungs, liver, brain, and bone) have often been limited to capillaries from non‐human species and visualized with restrictive imaging methods that could collapse the hydrated GCX (i.e., conventional TEM) with its composition often not being fully characterized.^84^, ^85^, ^86^ As earlier stated, the tight junctions and adherens junctions present in lung and brain capillaries suggest a strong barrier against cancer extravasation, so the GCX may play a role in mediating this process. Studies are ongoing in our lab on the GCX of human brain and lung microvascular ECs to characterize their GCX under static and physiological flow conditions utilizing immunofluorescence confocal microscopy. There is a need to fill this gap of knowledge to understand more about the mechanisms driving cancer extravasation.

To date, the studies examined the wholistic structure of the GCX with a wheat germ agglutinin lectin staining (primarily for sialic acid and secondarily for N‐acetylglucosamine which is found in HS and HA) along with individual fluorophores labeling HS, HA, and α2,6‐linked SA components of the endothelial GCX (Figures 4 and 5) (Okorafor et al., manuscript in preparation). The presence of these components has affected cancer cell attachment and extravasation of breast cancer cells.^44^, ^64^, ^65^ In one study, brain microvascular ECs cultured on glass slides were exposed to uniform shear stress conditions (12 dyn/cm^2^) for 6 h (Okorafor et al., manuscript in preparation). For the various GCX elements the following characteristics were quantified: mean fluorescence intensity which defines the aggregate density of the expressed GCX component,^82^ percent area coverage which measured the area coverage of the GCX component on the apical side of ECs, and thickness. The wholistic stain, WGA, showed almost 100% coverage for brain microvascular ECs (Figure 4D,E). When looking at individual components of the stained GCX, the results showed that α2,6‐linked SA residue in respect to percent area coverage was the most abundant GCX component for brain microvascular ECs. (Figure 4C,E). α2,6‐linked SA residue's mean fluorescence intensity (aggregate density) was also significantly higher compared to the other components (HA, HS) (Figure 4C,F). Thickness measurements were mostly comparable with the exception of HA showing decreased thickness compared to α2,6‐linked SA residue and HS (Figure 4C,G). Under shear stress, the HS component of brain EC GCX was concentrated at the endothelial junctions, suggesting a stronger barrier against cancer extravasation at junctions (Figure 4A).

**FIGURE 4:**
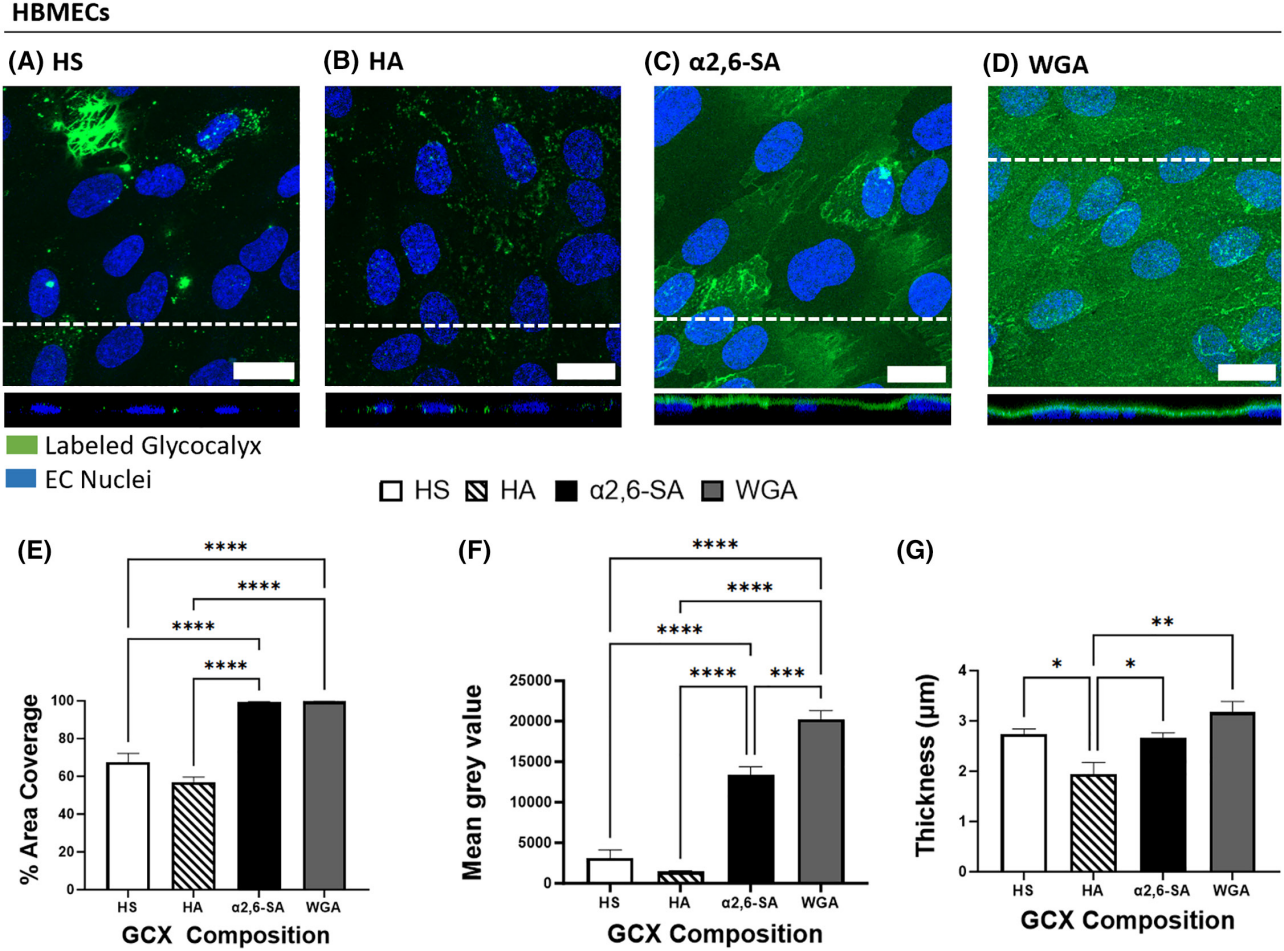
Human brain microvascular endothelial cells (HBMECs) exhibit notable elevation of sialic acid (SA) compared to other components of the GCX (Okorafor et al., manuscript in preparation). The data shown here comes from HBMEC monolayers grown on fibronectin‐coated glass substrates and subjected to 12 dyn/cm^2^ flow. Enface and orthogonal views of HBMECs were captured to visualize and computationally analyze distinct GCX components, including (A) heparan sulfate (HS), (B) hyaluronic acid (HA), (C) α 2‐6‐linked sialic acid (α 2‐6‐SA), and (D) whole GCX labeled with wheat germ agglutinin (WGA) lectin. Analysis of GCX components included (E) percent area coverage, (F) intensity, and (G) thickness conducted through computational analysis of the raw images. (However, it is important to mention that the image intensity settings were subsequently increased for the orthogonal views only, to make the components easier to distinguish by eye.) Results show mean ± SEM. Bar = 20 μm. Statistical analysis was assessed via one‐way ANOVA with post hoc Tukey analysis and is indicated by asterisks as follows: *p < .05, **p < .01, ***p < .001, and ****p < .0001. The sample size examined was *N* = 4.

**FIGURE 5:**
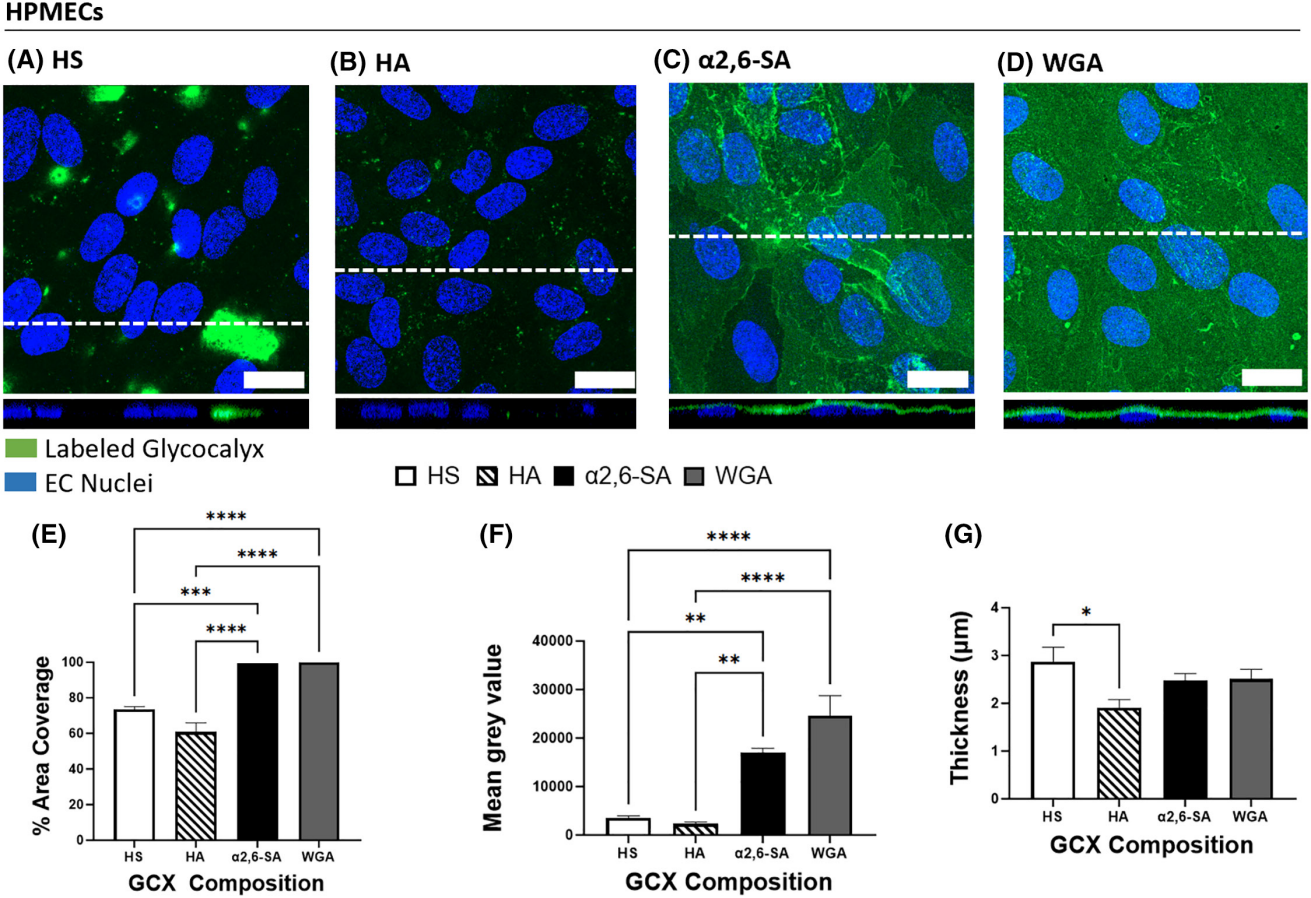
Human lung microvascular endothelial cells (HPMECs) exhibit notable elevation of sialic acid (SA) compared to other components of the GCX (Okorafor et al., manuscript in preparation). The data shown here comes from HPMEC monolayers grown on fibronectin‐coated glass substrates and subjected to 12 dyn/cm^2^ flow. Enface and orthogonal views of HPMECs were captured to visualize and computationally analyze distinct GCX components, including (A) heparan sulfate (HS), (B) hyaluronic acid (HA), (C) α 2‐6‐linked sialic acid (α 2‐6‐SA), and (D) whole GCX labeled with wheat germ agglutinin (WGA) lectin. Analysis of GCX components included (E) percent area coverage, (F) intensity, and (G) thickness conducted through computational analysis of the raw images. (However, it is important to mention that the image intensity settings were subsequently increased for the orthogonal views only, to make the components easier to distinguish by eye.) Results show mean ± SEM. Bar = 20 μm Statistical analysis was assessed via one‐way ANOVA with post hoc Tukey analysis and is indicated by asterisks as follows: *p < .05, **p < .01, ***p < .001, and ****p < .0001. The sample size examined was *N* = 4.

For lung microvascular ECs, GCX components behaved similarly to brain microvascular ECs under flow conditions. α2,6‐linked SA residue was the most abundant GCX component in respect to percent area coverage and mean fluorescence intensity for lung microvascular ECs compared to HA and HS (Figure 5A–C,E,F) (Okorafor et al., manuscript in preparation). Thickness measurements were mostly comparable with the exception of HA showing decreased thickness compared to HS. Under shear stress, similar to brain ECs, for lung ECs the HS GCX component concentrated at the endothelial junctions, indicating a strong barrier against cancer extravasation at junctions (Figure 5A). For the HA component, the mean intensity expression, percent area coverage, and thickness was low for lung microvascular ECs under flow conditions (Figure 5B,E–G).

Previously, most studies utilizing macrovascular ECs claimed HS to be the most prominent GCX component for ECs, making up 50%–90% of the GAGs, and claimed that the GCX is rich with HA.^61^, ^87^, ^88^, ^89^, ^90^, ^91^, ^92^ It is possible that microvascular ECs' GCX, particularly in organs that are common sites of TNBC metastasis, possess composition that differs from macrovascular ECs. The prominence of α2,6‐SA residue for these microvascular ECs may play a key role in CTC recruitment to endothelium since TNBC highly express CD22, a sialic‐acid‐binding immunoglobulin‐like lectin, that preferentially binds to α2,6‐linked SA residue.^93^, ^94^ More work needs to be done in characterizing the GCX of these microvessel ECs on a more physiological matrix.

## 4 FUTURE DIRECTIONS

The focus on TNBC in this review is driven by its unique characteristics, extensively studied to understand its highly aggressive behavior and propensity for secondary tumor formation. Despite the extensive investigation into TNBC's molecular and genetic profile,^95^, ^96^, ^97^, ^98^ there has been limited exploration of the contribution of mechanical factors such as shear stress, interstitial flow, and ECM stiffness to TNBC cell extravasation. Nevertheless, literature suggests that TNBCs are particularly responsive to mechanical signals compared to other breast cancer subtypes such as luminal A, luminal B, and HER2‐positive, underscoring the importance of addressing this gap in synthesized knowledge.^47^, ^64^, ^99^

While this review fills a critical gap in our understanding, it would be valuable to investigate whether the mechanical mechanisms described herein apply to the metastasis of other breast cancer subtypes, as well as different cancer cell types such as lung, colon, and prostate, which exhibit significant differences in their metastatic characteristics.^11^, ^25^, ^100^ Expanding the scope of future exploration to encompass a broader range of cancer cell types is essential for fully comprehending how mechanics regulate metastasis.

The impact of the mechanical environment on TNBC extravasation is greatly understudied, especially in regard to the role that the subendothelial matrix stiffness plays in regulating this process. Stiffness may play a key role in guiding cancer cells to secondary sites, as seen in fibrotic lung conditions, through its regulation of the endothelial GCX. Although normal lung possesses low stiffness matrix (1–5 kPa), the fibrotic lung is characterized by its high matrix stiffness (20–100 kPa) which may affect endothelial GCX's structure and susceptibility toward cancer extravasation.^101^ We propose utilizing a flow chamber model that precisely regulates the shear stress induced from flow and mechanically tunable hydrogels^82^ to match in vivo healthy and pathological conditions (fibrosis) to determine the effect these mechanical factors have on GCX expression. We hypothesize that a high matrix stiffness environment will inhibit overall GCX expression while increasing α2,6‐linked SA expression relative to other GCX components, compromising the vascular permeability barrier and increasing the tendency for CTC extravasation via the α2,6‐linked SA interaction with CD22 that is bound to the CTC surface. One challenge to understanding the role GCX plays in mediating this process is its visualization. The visualization of microvascular ECs especially from popular metastatic sites of TNBC, lungs, have often been limited to non‐human species and used methods that may collapse the fragile, hydrated structure. Developing a visualization method that can overcome the challenge of GCX fragility and preserve the hydrated structure of the GCX, will make it possible to fully uncover the GCX composition at these frequent sites of metastasis and move the field forward. In addition, we would like to clarify SA's role in CTC extravasation since two previously mentioned studies showed contradiction. Our previous data has shown that α2,6‐linked SA is a prominent component of endothelial GCX for lung microvascular ECs. SA expression could promote recruitment of cancer cells to endothelium through CD22 interaction with SA. Determining whether SA blockage in the fibrotic lung environment inhibits CTC extravasation will provide a potential anti‐metastasis treatment option. TNBC is a deadly disease with limited treatment options so finding new vascular targets will be crucial to limiting its metastatic cascade.

## AUTHOR CONTRIBUTIONS

Chinedu C. Okorafor wrote the original paper draft. Chinedu C. Okorafor and Sanjana Shastri collected the data, designed, and performed the analysis. Ke Wen developed the code for percent area coverage analysis and performed the analysis. Chinedu C. Okorafor and Sanjana Shastri prepared the figures. Eno E. Ebong provided resources to support the research, supervised the project, and reviewed and edited the manuscript.

## FUNDING INFORMATION

This work was primarily funded by the National Science Foundation (NSF) CAREER Award CMMI 1846962 (to E. E. Ebong). Additional funding was provided by the NSF Louis Stokes Alliance for Minority Participation Bridge to the Doctorate (LSAMP‐BD) Strategic Advancement of Rising Scholars (STARS) Award HRD 1812412 (to Northeastern University; C. Okorafor was nominated by Northeastern University LSAMP and supported as a predoctoral fellow on this award).

## DISCLOSURES

The authors declare that the research was conducted in the absence of any commercial or financial relationships that could be construed as a potential conflict of interest.

## Data Availability

The data that support the findings of this study are available in the article.
